# Activation characteristics of Ty3-retrotransposons after spaceflight and genetic stability of insertion sites in rice progeny

**DOI:** 10.3389/fpls.2024.1452592

**Published:** 2024-12-02

**Authors:** Qing Yang, Lishan Chen, Meng Zhang, Wei Wang, Binquan Zhang, Dazhuang Zhou, Yeqing Sun

**Affiliations:** ^1^ Institute of Environmental Systems Biology, College of Environmental Science and Engineering, Dalian Maritime University, Dalian, China; ^2^ National Space Science Center, Chinese Academy of Sciences, Beijing, China

**Keywords:** Ty3-retrotransposons, space HZE particles, DNA methylation, spaceflight, rice

## Abstract

**Introduction:**

The space environment is mutagenic and may induce genomic and phenotypic variations. Exploring the changes in transposon activity in the rice genome under space radiation is of great significance.

**Methods:**

To analyze the activation characteristics of Ty3-retrotransposons and genetic stability of insertion sites in rice progeny after spaceflight, seeds of Nipponbare, DN416, and DN423 were exposed on board the SJ-10 recoverable satellite for 12.5 days. The differential methylation and transcription levels of Ty3-retrotransposons in the genome of Nipponbare's F0 generation after spaceflight, as well as the genetic stability of Ty3-retrotransposon insertion sites in DN416 and DN423 from F3 to F5 generations, was analyzed.

**Results:**

The study found that the retrotransposons of ancient and young transposon families underwent demethylation from the tillering to heading stages of Nipponbare plants, which were F0 generation of space-exposed seeds, when the Nipponbare seeds were hit by single space high charge and energy (HZE) particles with LET ≥ 100 keV/μm. the transcription levels significantly increased in ancient transposon families (osr30, osr40, and rire10) and young transposon families (dagul, rn215-125, osr37, RLG_15, osr34, rire8, rire3, rire2, and hopi) (p ≤ 0.05) when LET > 100 keV/μm. Furthermore, the young Ty3-retrotransposons, which included the hopi, squiq, dasheng, rire2, rire3, rire8, osr34, rn_215-125, dagul, and RLG_15 families, underwent 1 to 8 transpositions in the F3 to F5 of DN416 and DN423 mutants, and some of these transposon insertion sites were stably inherited.

**Discussion:**

The research holds great significance for understanding the activation characteristics of Ty3-retrotransposons in the rice genome induced by space radiation and the genetic characteristics of transposon insertion sites in its progeny.

## 1 Introduction

Space environment is a complex abiotic stress setting characterized by microgravity and strong radiation. The space radiation is primarily composed of Galactic Cosmic Rays (GCR), Solar Particle Events (SPE), and Trapped Belt Radiation, and it can induce genetic mutations in organisms within near-Earth orbit spacecraft (Yu et al., 2007; Long et al., 2009; Ou et al., 2009; Yatagai et al., 2011; Shi et al., 2014, 2017; Li et al., 2020; Jin et al., 2022). The space radiation field inside the spacecraft consisted of space HZE particles and secondary particles generated by their penetration through the spacecraft walls. For example, in the SJ-10 retrievable satellite, which flew at an altitude of 252 km, the dose equivalent of space high-energy particle radiation inside the cabin accounts for 60% of all radiation (Zhou et al., 2018). The research using retrievable satellites carrying plant seeds showed that exposure to the space environment induced changes in the epigenetic levels of the rice genome, and the extent of these changes was significantly positively correlated with the genome mutation rate (Shi et al., 2014). Space environment could also alter cytosine methylation patterns and activate transposons (Ou et al., 2009; Xu et al., 2018). And these activated transposons could transpose, regulate genome size and gene function, and be inherited by the next generation (Long et al., 2009; Li et al., 2020; Jin et al., 2022). These studies indicated that genetic mutations induced by the space environment may be related to the activation of transposons.

Transposons were classified into DNA transposons and RNA retrotransposons based on their transposition mechanisms. DNA transposons transpose through a “cut-and-paste” mechanism, while RNA retrotransposons transpose by a “copy-and-paste” mechanism. DNA transposons were primarily located upstream and downstream of functional genes, and they could participate in the regulation of functional genes. RNA retrotransposons were primarily located in centromeric regions, which played a crucial role in chromosome segregation (Lippman et al., 2004; Dawe and Henikoff, 2006; Bennetzen et al., 2012; Tsukahara et al., 2012; Bennetzen and Wang, 2014; Sigman and Slotkin, 2016). The insertion of RNA retrotransposons could lead to chromosomal instability and potentially cause non-disjunction or mis-segregation of chromosomes, which resulted in abnormal cell division (Burrack and Berman, 2012). In angiosperms, genome size was positively correlated with transposon content (Negi et al., 2016; Mbichi et al., 2020). Zhang et al. discovered that the rapid expansion of LTR retrotransposons was a significant driving force in the lineage-specific evolution of the rice AA genome. This expansion not only shaped the structure of the rice genome to varying degrees but also facilitated the speciation and diversification of rice (Zhang and Gao, 2017). Daron et al. analyzed the transposons in the 3B chromosome of barley and found that the expansion of the 3B chromosome was closely related to the episodic bursts of retrotransposons (Daron et al., 2014). Therefore, in this study, the most abundant retrotransposon, Ty3-retrotransposon, was chosen as the research subject in the rice genome.

Previous studies have shown that spaceflight can induce genomic transposon instability. In order to investigate whether space HZE particles in the space radiation environment are the primary triggers for activating Ty3-retrotransposons and to understand the genetic stability changes in these transposon families after activation. The rice seeds were carried by SJ-10 recoverable satellite, which flew at an altitude of 252 km for 12.5 days. The satellite received cosmic rays from the South Atlantic Geomagnetic Anomaly (SAA), a region with magnetic field anomalies in the southern part of South America and the South Atlantic Ocean, and the magnetic field was 30% to 50% weaker than that in other areas at the same latitudes (Gee and Kent, 2007; Hare et al., 2018; Schuch, 2019; Yu et al., 2023). Rice seeds may be hit by space HZE particles when SJ-10 pass through the SAA, and the LET values of space HZE particles was measured by CR-39 nuclear track detectors (Zhou et al., 2018). After returning to the Earth, the rice seeds hit by individual space HZE particles were planted and sequenced. Subsequently, the activity changes of intact Ty3-retrotransposons in the rice genome were analyzed after the seeds were exposed to space HZE particles with different LET (linear energy transfer), as well as the mutagenesis patterns in their progeny.

In this study, the Ty3-retrotransposons of the Nipponbare genome were classified into different transposon families, and they were further divided into ancient and young transposon families based on these insertion times. Subsequently, the study analyzed the differential levels of methylation and expression of ancient and young Ty3-retrotransposon in the F0 generation of space-exposed seeds. Finally, the transposon insertion polymorphisms (TIPs) of activated Ty3-retrotransposons were analyzed in the F3-F5 generations of space mutagenic lines SA3-7, SA6-2, and SC6-6. The results showed when rice seeds were exposed to space HZE particles with LET > 100 keV/μm, Ty3-retrotransposons could be more effectively activated through demethylation in the F0 generation of space-exposed seeds. In addition, the retrotransposons activated by space radiation underwent transposition in the progeny, and some insertion sites gradually stabilized. The study helped in understanding the mechanisms of space radiation-induced mutagenesis in plant seeds and the role of transposons in rice genetic variation and differentiation. It also provided deeper insights into the relationship between the space environment and the diversification of angiosperms.

## 2 Materials and methods

### 2.1 Plant materials

Rice (*Oryza. Sativa* L. spp. *Japonica*, var *Nipponbare*) was utilized as a model plant in this study. The seeds of Nipponbare, Dongnong416 (DN416), and Dongnong423 (DN423) were carried by the SJ-10 retrievable satellite at an altitude of 252 km with an inclination of 42 degrees for a duration of 12.5 days. The satellite received cosmic rays from the South Atlantic Geomagnetic Anomaly (SAA), a region with magnetic field anomalies in the southern part of South America and the South Atlantic Ocean, and the magnetic field was 30% to 50% weaker than that in other areas at the same latitudes (Gee and Kent, 2007; Hare et al., 2018; Schuch, 2019; Yu et al., 2023). Therefore, Rice seeds may be hit by space HZE particles when SJ-10 pass through the SAA region (Zhou et al., 2018).

The rice seeds were placed in detection stacks made of the nuclear track detection material CR39, and the linear energy transfer (LET) values of space HZE particles were accurately calculated (Zhou et al., 2018). Three ground control groups and eight spaceflight groups were selected from the Nipponbare. The spaceflight groups included the seeds that were not hit by HZE particles (RN) and the seeds hit by HZE particles with different LET values, specifically in the range of 52.7-186.1 KeV/μm (**Supplementary Table S1**). The three ground control seeds and eight spaceflight seeds were simultaneously planted in phytotron for the full growth cycle, and rice leaves were collected during the tillering and heading stages in the F0 generation of space-exposed seeds.

To analyze the genetic effects of activated transposons on offspring exposed to space radiation, three space mutagenic lines from DN423 and DN416 were selected for further study, including SA3-7 with increased plant height and long awn of DN423, SA6-2 with increased tillering number and SC6-6 with reduced tillering number of DN416 (**Supplementary Table S2**).

The plants mutated by space radiation were identified and named for each individual plant in the F0 generation. These mutagenic plants and their corresponding ground control plants were tracked and planted by using the pedigree method. In order to ensure the simultaneous analysis of successive generations of bio-materials, 15 plants of each of the three space mutagenic lines and corresponding ground control plants of the F2-F4 generations were planted in a phytotron at the same time. The F3-F5 generations of space mutagenic plants were simultaneously obtained and applied in this study (**Supplementary Figure S1**). The main variable traits were as follows: the space-induced increase in plant height, accompanied by long awns, was observed in SA3-7, which maintained from the parental generation to the F5 generation. The space induced increase in the tillering phenotype was observed in SA6-2 and gradually recovered in offspring. The reduced tillering number phenotype of SC6-6 was identified in the F1 generation, and the mutant phenotype was maintained stably until the F5 generation.

Rice plants were cultivated using the hydroponic method in a phytotron under controlled conditions: 28°C/25°C (day/night), 70% humidity, light intensity of 300 μmol·m^-2^·s^-1^, and a 14-hour light period. The hydroponic nutrient solution was prepared with commercially available YOSHIDA rice nutrient solution (Coolaber) and pure water. The rice was grown through its entire life cycle, and leaves were collected at the tillering and heading stage, immediately flash-frozen in liquid nitrogen, and stored at -80°C for future analysis.

### 2.2 Whole-genome bisulfite DNA sequencing

the total genomic DNA was isolated from rice leaves for each rice plant sample. A total of 5.2 mg genomic DNA was fragmented by sonication to 200-300 bp with a Covaris S220. Then, these DNA fragments were treated twice with bisulfite using an EZ DNA Methylation-GoldTM Kit (Zymo Research) according to the manufacturer’s instructions. The library concentration was quantified by Qubit^®^ 2.0 Flurometer (Life Technologies, CA, USA) and the results were quantified via quantitative PCR, and the insert size was assayed on Agilent Bioanalyzer 2100 system. The library preparations were sequenced on an Illumina Hiseq X ten platform, and 125bp/150bp paired-end reads were generated. Bismark software (version 0.16.3) was used to perform alignments of bisulfite-treated reads to a reference genome (-X 700 –dovetail) (Andrews, 2011).

### 2.3 RNA sequencing

For each rice plant of Nipponbare, a total of 3 μg RNA per sample (leaf) was used as input material for the RNA library preparations. Sequencing libraries were generated using the NEBNext^®^ Ultra™ RNA Library Prep Kit for Illumina^®^ (NEB, USA) following the manufacturer’s recommendations. The library preparations were sequenced on an Illumina Hiseq platform and 125 bp/150 bp paired-end reads were generated. Clean RNA sequencing data were mapped to each genome using hisat2 (v2.1.0) (Kim et al., 2015).

### 2.4 Whole-genome resequencing

the Nipponbare and space mutagenic lines SA3-7, SA6-2 and SC6-6 were performed via whole-genome resequencing on the Illumina platform. A total of 0.2 μg DNA per sample (leaf) was used as input material for the DNA library preparations. Sequencing library was generated using NEB Next^®^ Ultra™ DNA Library Prep Kit (Nipponbare) and TruSeq DNA PCR-free prep kit (DN416 and DN426) for Illumina following manufacturer’s recommendations. Sequencing reads were aligned to the reference genome using BWA (Li and Durbin, 2009) with default parameters. Subsequent processing, including duplicate removal was proformed using samtools and PICARD (http://picard.sourceforge.net).

### 2.5 The library of long terminal repeat retrotransposons (LTR-RTs)

The LTR-RTs database of rice were constructed using the LTR_retriever in this study (Ou and Jiang, 2018). 1667 Ty3-retrotransposons were identified in the rice Nipponbare reference genome. The Ty3-retrotransposons were categorized based on several criteria, including LTR sequence or protein-coding sequence identity exceeding 70%, the overlap sequence surpassing 80 bp, and a mutual overlap of 80% in their lengths. 90 of these families contained more than one transposon (Wicker et al., 2007). The name of each family was determined by referring the RetrOryza transposon database (Cristian et al., 2007; Choi and Purugganan, 2018). The families not present in RetrOryza were named using the format “RLG + number”, for example, “RLG_0”. There are 21 transposon families within the Ty3-retrotransposon superfamily, each with a copy number exceeding 18 (**Supplementary Table S3**).

### 2.6 Statistical analysis and significance calculation of Ty3-retrotransposon methylation levels

Each Ty3-retrotransposon was divided into five parts based on its structural composition: the left LTR, protein-coding/internal region, right LTR, and 2000 bp upstream and downstream of the transposon. Each of these regions were further divided into 50, 30, 150, 30, and 50 bins, for a total of 310 bins. The mean methylation level (MML_bin_) of each bin, based on the methylation level of cytosine (C), was calculated as follows:


$$MML_{bin}=\frac{mC}{mC+umC}$$

MML_bin_ represents the methylation level, and mC and umC denote the reads counts of methylated C and unmethylated C, respectively.

The Wilcoxon rank-sum test (WRST) was employed to compare the differences in methylation levels among different transposons.

### 2.7 Calculation of transposable element insertion time

To calculate the insertion time of each LTR-RT, this study first compared the divergence of the left and right LTRs to determine the LTR evolutionary distance (K). Then, the LTR evolutionary distance and the divergence substitution rate were used to calculate the insertion time of the LTR-RT. The specific formula is as follows (Kimura, 1980).


$$T=\frac{K}{2\gamma }$$

For the DNA evolutionary distance (K), for two DNA sequences, A and B, where all mutations during evolution are independent, the probability of change at each position is the same, and mutations at A, T, C and G are random, with no insertions or deletions occurring. The sequence divergence rate between A and B is denoted as p, representing the proportion of differing bases between the two sequences. And the evolutionary distance (K) between A and B is calculated as follows (Jukes and Cantor, 1969):


$$K=-\frac{3}{4}\times \text{ln}\;\left(1-4\times \frac{p}{3}\right)$$

The γ representing the natural mutation rate of transposon regions in rice. the mutation rate per nucleotide site in the genomes of grasses is 6.5×10^-9^ substitutions/site/year. And the nucleotide mutation frequency of transposable elements in rice is twice that of the normal genome, at 1.3×10^-8^ substitutions/site/year (Ma and Bennetzen, 2004; Daron et al., 2014).

### 2.8 Identification of TE insertion sites

The soft-clipped sequences were extracted from the alignment results to confirm the presence of new transposon insertion sites. The sequences corresponding to the positive or negative strands of the soft-clip sequences were extracted. Subsequently, the newly extracted sequences were re-aligned to the reference genome by using BWA (mem -t 4 -k 32 -M). The positions with target site duplication (TSD) was recorded after the realignment. Subsequently, the sequences were aligned to Ty3-retrotransposons. The transposon might have inserted into a new site if the sequences were aligned to both ends of the element (**Supplementary Figure S2**). In this study, to validate the model’s reliability, 20 transposons were randomly selected from the established LTR-RT database and then randomly inserted into 100 positions on chromosome 3 of rice. The simulations of paired-end reads for all simulated chromosome 3 datasets were conducted using pIRS (simulate –l 100 –x 15) (Fan, 2012). For each dataset, simulated sequence reads were generated at 15x coverage depth. Subsequently, all insertion sites on chromosome 3 and the names of inserted transposons were obtained through sequence alignment. It was found that 82% of the sites could be accurately located by comparing the predicted values with the actual values, with identifiable transposon names detected at 79% of the insertion sites. the code for the ITEIS was stored in GitHub (https://github.com/yq342/ITEIS).

## 3 Results

### 3.1 Evolutionary characteristics of Ty3-retrotransposons in Nipponbare

As RNA retrotransposons transpose through a copy-and-paste mechanism, the activation and transposition of RNA retrotransposons were closely associated with genome size. In order to investigate the correlation between RNA retrotransposons and rice genome expansion, the intact LTR-RTs were extracted from the Nipponbare reference genome by utilizing LTR_retriever in rice (Ou and Jiang, 2018). A total of 2234 full-length LTR-RTs were identified in rice. There were 1667 Ty3-retrotransposons and 567 other LTR retrotransposons, with the quantity of Ty3-retrotransposons being roughly 2.9 times higher than that of other LTR retrotransposons. The relationship between the burst amplification of Ty3-retrotransposons and the expansion of the rice genome appeared stronger. Therefore, Ty3-retrotransposon was chosen for analysis in this study. the 1667 Ty3-retrotransposons, 14 could not have their insertion time calculated and were deleted. The remaining retrotransposons were categorized into 176 families based on criteria such as LTR sequence or protein-coding sequence identity exceeding 70%, overlapping sequences surpassing 80 bp, and a mutual overlap of 80% in their lengths (Cristian et al., 2007; Wicker et al., 2007). 86 of these families contained more than one transposon (**Supplementary Table S3**). The LTR regions of Ty3-retrotransposons were aligned using BLAST. The insertion times of LTR-RTs were estimated based on nucleotide mutation rates (Kimura, 1980; Ma and Bennetzen, 2004). A total of 8 families originated approximately 3.5 million years ago (MYA), while 168 families originated 2.6 MYA for the Ty3-retrotransposons.

**Figure 1:**
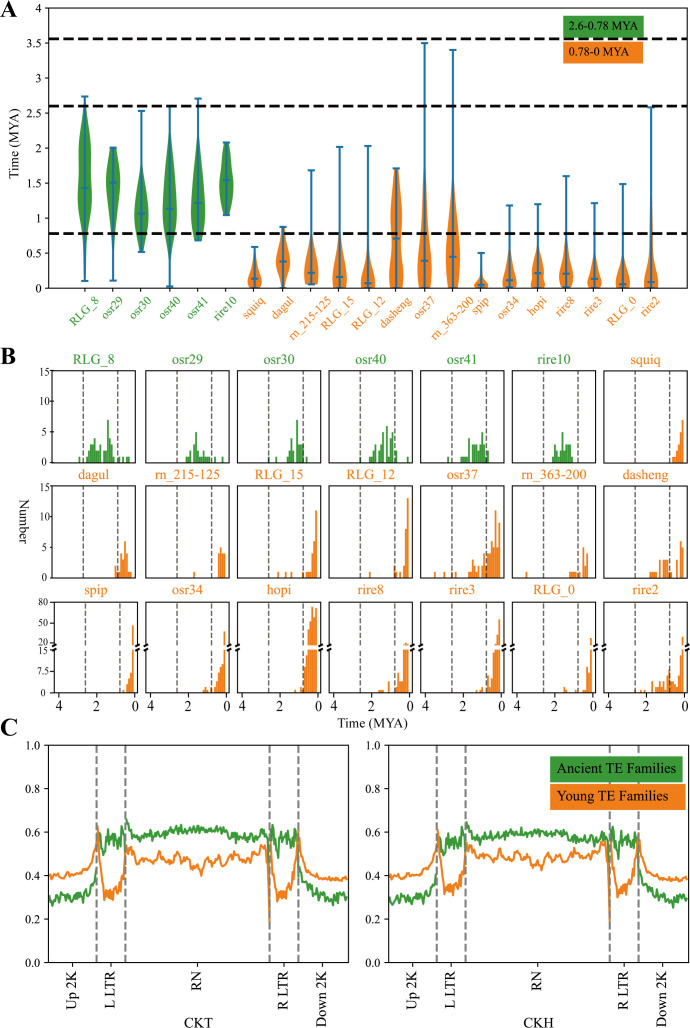
The insertion time of Ty3-retrotransposons in rice and the means methylation level in ancient and young Ty3-retrotransposons family. **(A)** showed the burst expansion times of Ty3-retrotransposons. The x-axis in the figure represents different transposon families, while the left y-axis represents the time of transposon insertion (MYA). **(B)** illustrated the number of new retrotransposon insertions for different TE families every 0.1 million years. The vertical dashed lines represent 2.6 MYA and 0.78 MYA, respectively. **(C)** showed the average methylation levels in the 2-kb upstream and downstream regions of TEs, long terminal repeats (LTRs), and internal regions for both ancient and young transposon families within the 21 Ty3-retrotransposon families. Green indicates ancient transposon families and yellow indicates young transposon families.

In this study, 21 Ty3-retrotransposon families were chosen, each of which had a copy number exceeding 18, encompassing 81.2% of the total (**Figure 1A**; **Table 1**). The Ty3-retrotransposons predominantly originated after 3.5 MYA and experienced two burst expansions after 2.6 MYA and 0.78 MYA (Leonardo et al., 2016). The first burst occurred at 2.6-0.78 MYA (ancient family), which included 6 TE families (**Table 1**). The burst features of ancient family gradually expanded from 1 to 21-39, followed by gradual re-silenced. The second burst occurred after 0.78 MYA (young family), and included 15 TE families (**Table 1**). Each family contained a significantly greater number of transposons, ranging from 18 to 361 (**Figure 1B**).

**Table 1:** The burst times of Ty3-retrotransposon families with copy numbers greater than 18.

Time (MYA)	Ty3-retrotransposon family
2.60 - 0.78	RLG_8, rire10, osr41, osr40, osr30, osr29
0.78 – 0.00	hopi, osr34, dagul, RLG_12, RLG_15, rire8, RLG_0, rire3, spip, rire2, osr37, dasheng, squiq, rn_215-125, rn_363-200

The mean methylation levels of ancient and young Ty3-retrotransposons were calculated at the tillering (CKT) and heading (CKH) stages for the ground control group. The results indicated that, in both growth stages, the methylation levels of ancient transposon families exceeded those of young transposon families in both the LTR and protein-coding/internal region regions (Wilcoxon rank sum test, p ≤ 0.05), but the trend was reversed in the upstream and downstream 2 kb sequences of transposons (**Figure 1C**).

### 3.2 Differential methylation levels of Ty3-retrotransposon families in Nipponbare hit by space HZE particles with different LETs

The study collected eight spaceflight seeds, including one seed that were not hit by HZE particles (RN) and seven seeds that were hit by HZE particles with different LET values, specifically ranging from 52.7 to 186.1 KeV/μm (**Supplementary Table S1**). These seeds were planted in the phytotron, and the rice leaves were collected during the tillering and heading stages for the F0 generation of space-exposed seeds. To analyze the changes in methylation levels of different Ty3-retrotransposon families in the Nipponbare after being hit by space HZE particles with different LETs (specific details are provided in Materials and Methods), This study counted the number of Ty3-retrotransposon with significant changes in methylation levels (Wilcoxon rank-sum test, p ≤ 0.05) in 21 retrotransposon families during the tillering and heading stages in the eight spaceflight groups for the F0 generation of space-exposed seeds.

The study revealed for the ancient transposon families, compared to the control group, in the osr29, osr40, osr41 and rire10 families, containing 23, 37, 33 and 26 transposons, the average proportion of hyper-methylated TEs decreased from 1.6%, 22.6%, 25.0% and 13.5% to 1.1%, 2.0%, 1.9% and 0.0%, respectively, from the tillering stage to the heading stage across all treatment groups. in the osr29, osr30, osr40, osr41 and rire10 families, the proportion of demethylated transposons increased from 1.1%, 2.2%, 2.4%, 4.2% and 1.9% to 11.4%, 30.8%, 40.5%, 30.3%, 43.8%, respectively.

For the young transposon families, compared to the control group, in the dagul and rn_215-125 families, containing 24 and 18 transposons, the average proportion of hyper-methylated TEs decreased from 35.9% and 8% to 4.1% and 2.0%, respectively, from the tillering stage to the heading stage across all treatment groups. the proportion of demethylated transposons increased from 1.0% and 0% to 6.2% and 17.4%, respectively. in the RLG_12 and spip families, containing 33 and 57 transposons, the average proportion of hyper-methylated TEs decreased from 9.8% and 15.1% to 5.3% and 7.7%, respectively, from the tillering stage to the heading stage across all treatment groups. the proportion of demethylated transposons increased from 16.7% and 12.1% to 18.9% and 16.4%, respectively. The rn_363-200, RLG_0 and osr37 families, containing 18, 53 and 71 transposons respectively, the average proportion of hyper-methylated TEs decreased from 0.7%, 5.0% and 0.9% to 0.2%, 1.1% and 0.5% from the tillering stage to the heading stage across all treatment groups. And in the RLG_0 and osr37 families, the proportion of demethylated transposons increased from 13.7% and 7.0% to 17.5% and 9.5% respectively. conversely in the rn_363-200 families the proportion of demethylated transposons decreased from 23.6% to 17.4%. the RLG_15, osr34, rire8, rire3, rire2 and hopi families, containing 30, 74, 76, 135, 147 and 361 transposons respectively, the average proportion of hyper-methylated TEs decreased from 23.8%, 27.7%, 13.8%, 24.1%, 25.9% and 21.7% to 7.9%, 10.3%, 3.8%, 9.7%, 7.7% and 10.7% from the tillering stage to the heading stage across all treatment groups. the proportion of demethylated transposons increased from 2.1%, 10.9%, 5.3%, 8.4%, 6.2% and 7.8% to 7.1%, 19.5%, 12.8%, 22.7%, 23.6% and 17.9% respectively.

Further analysis of the methylation levels of different Ty3-retrotransposon families in different LET treatment groups revealed that during the heading stage, the ancient families osr29, osr30, osr40, osr41 and rire10 underwent demethylation in RNH2, R60H2, R60H3, R150H1, R200H1 and R200H2 group, the young transposon families dagul and rn_215-125 families underwent demethylation in R150H1, R200H1 and R200H2 group in the F0 generation of space-exposed seeds. In addition, rn_363-200, RLG_0, osr37, RLG_0, osr37, RLG_15, osr34, rire8, rire3, rire2 and hopi underwent demethylation in R100H2, R00H3, R150H1, R200H1 and R200H2 group in the F0 generation of space-exposed seeds.

**Figure 2:**
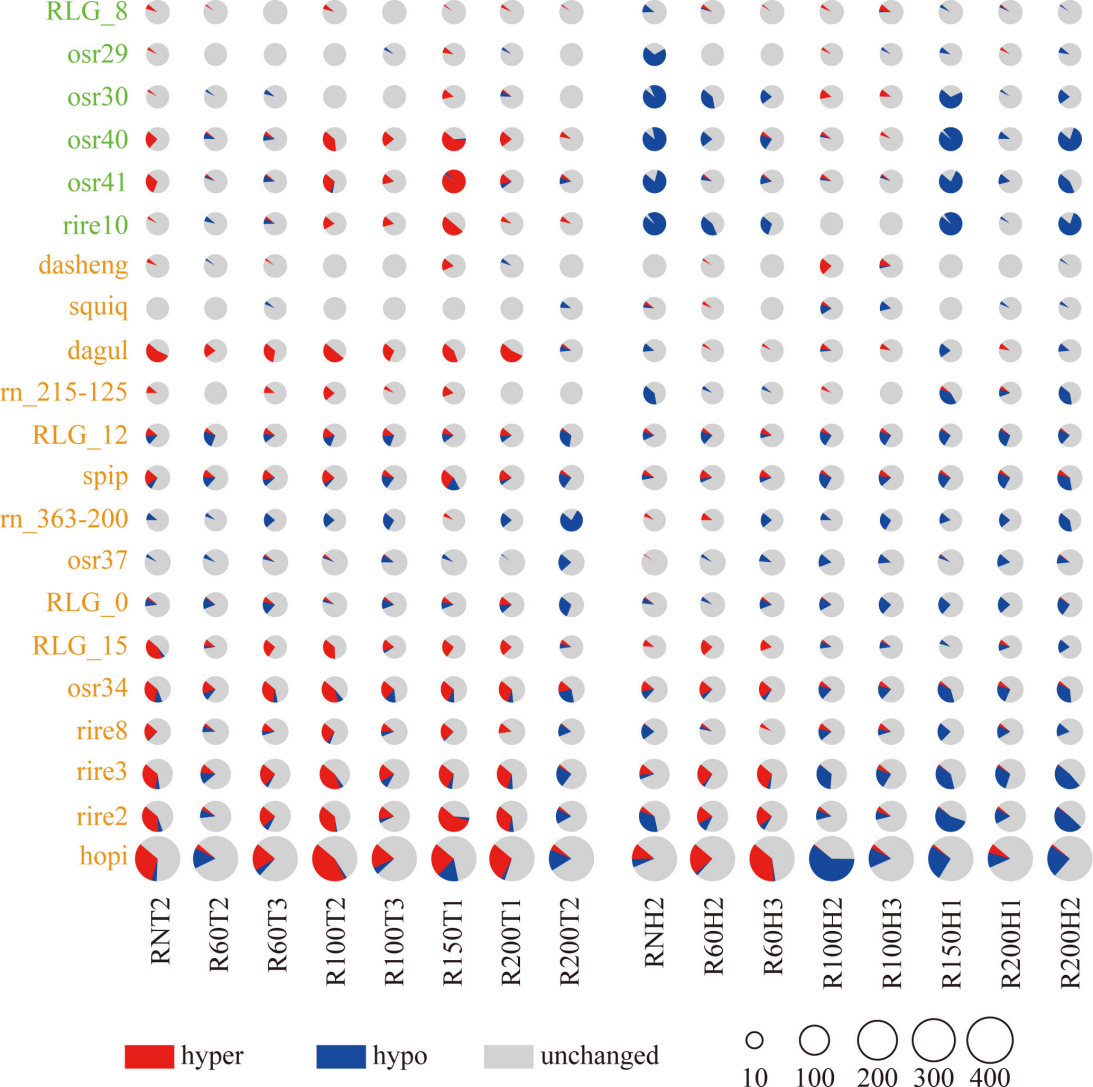
The number of hypermethylated and hypomethylated in varied transposon families among different groups. The x-axis represents the different LET treatment groups at the tillering (T) and heading (H) stages. The y-axis represents transposon families, with green indicating ancient transposon families and yellow indicating young transposon families. In the graph, red indicates the number of hypermethylated transposons, blue indicates the number of hypomethylated, and gray indicates the number of unchanged transposons.

The results indicated that after spaceflight, the rice seeds, whether not hit by space HZE particles (RN) or hit by space HZE particles with different LET, exhibited changes in the methylation levels of both ancient and young Ty3-retrotransposon families in the rice genome for the F0 generation (**Figure 2**; **Supplementary Table S4**). Additionally, from the tillering stage to the heading stage, the number of demethylated Ty3-retrotransposon gradually increased, particularly in some young transposon families, when rice seeds were hit by space HZE particles with LET ≥ 100 keV/μm.

### 3.3 Differential expression levels of Ty3-retrotransposon families in Nipponbare hit by space HZE particles with different LETs

Previous studies demonstrated that the methylation levels of Ty3-retrotransposons in the rice genome with exhibited dynamic changes at different developmental stages in the F0 generation when rice seeds were exposed to space HZE particles. Moreover, when the LET ≥ 100 keV/μm, the young retrotransposons, containing RLG_12, spip, rn 363-200, RLG_0, osr37, RLG_15, osr34, rire8, rire3, rire2 and hopi, underwent demethylation changes during the heading stage. To investigate the activity of demethylated retrotransposons, the study utilized TEtranscripts (Jin et al., 2015) to calculate the differential expression levels of Ty3-retrotransposons in the genomes of various groups at tillering stage and heading stage in the F0 generation of space-exposed seeds (**Supplementary Figure S3**).

**Figure 3:**
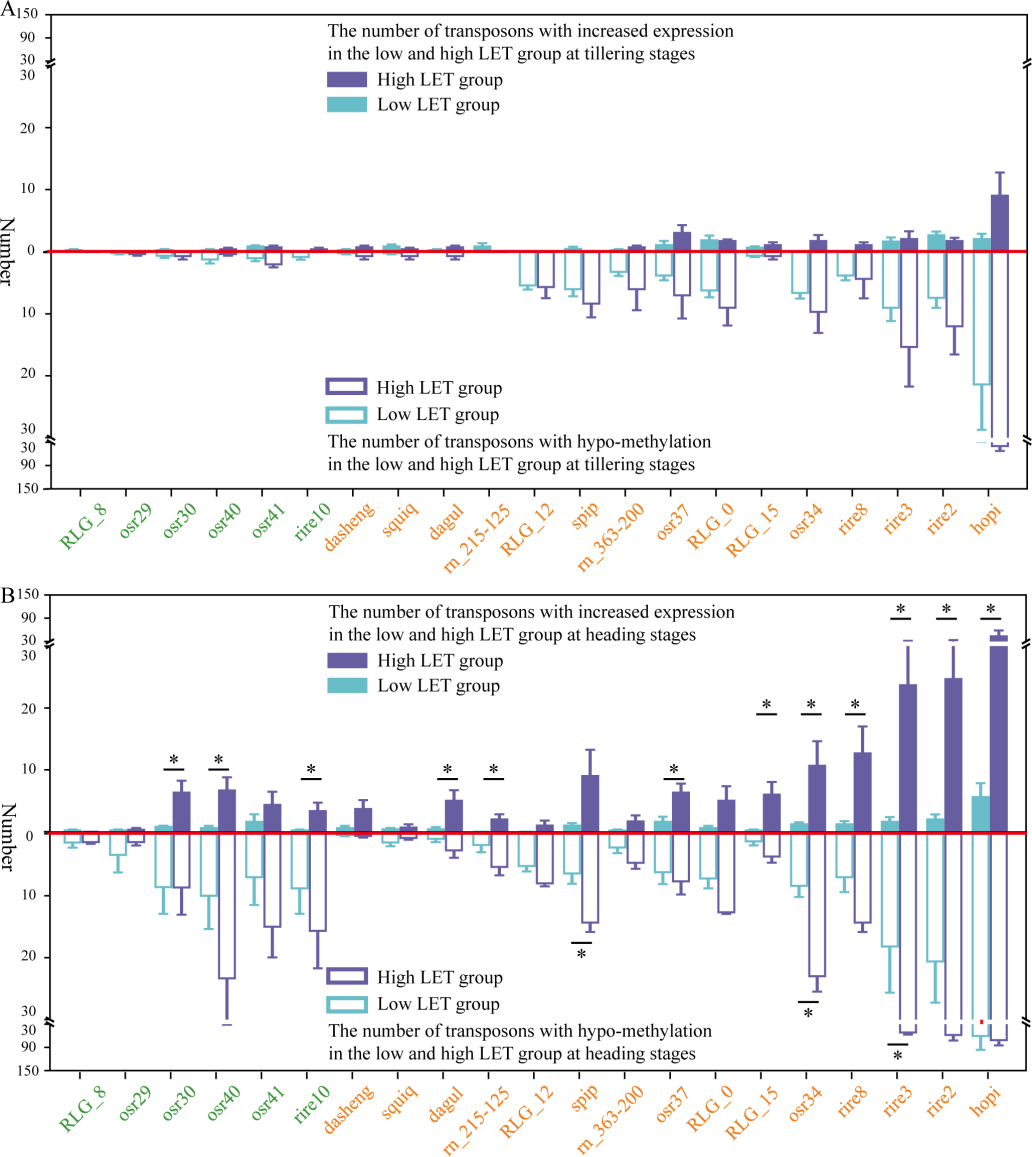
The number of demethylated and increased transcription retrotransposons in different transposon families across high (LET > 100keV/µm) and low (LET ≤ 100keV/µm) LET group at tillering and heading stages. **(A)** represents at the tillering stage, **(B)** represents at the heading stage, and “*” represents P-value ≤ 0.05.

The study found that the increased transcription of retrotransposons primarily occurred during the heading stage in the F0 generation of space-exposed seeds. While the methylation levels of retrotransposons decrease when rice seeds were exposed to space HZE particles with LET ≥ 100 keV/μm, significantly increased transcription of retrotransposons (P ≤ 0.05) was observed exclusively in the groups where LET > 100 keV/μm (**Figure 3**). Therefore, the groups hit by space HZE particles were divided into high LET (LET > 100 keV/μm) and low LET (LET ≤ 100 keV/μm) groups. The low LET group included 5 samples, while the high LET group included 3 samples (**Supplementary Table S1**). The ground control group consisted of 3 samples. This study first quantified the number of retrotransposons undergoing demethylation and enhanced expression in different transposon families for each group in the high LET group and low LET group compared to the ground control group in the F0 generation of space-exposed seeds. A T-test (P-value ≤ 0.05) was then used to assess whether there were significant differences in the number of demethylated and increased transcription retrotransposons between the high and low LET groups in the F0 generation of space-exposed seeds.

Further analysis found that during the tillering stage, there was no significant difference in transposon activity between the high and low LET groups in the F0 generation of space-exposed seeds (**Figure 3A**). However, at the heading stage, the number of increased transcription retrotransposons in ancient transposon families (osr30, osr40, and rire10) and young transposon families (dagul, rn215-125, osr37, RLG_15, osr34, rire8, rire3, rire2, and hopi) was significantly higher in the high LET group compared to the low LET group (P ≤ 0.05). Specifically, the number of transcribed Ty3-retrotransposons in the young retrotransposon families osr34, rire8, rire3, rire2, and hopi ranged from 11 to 43. This suggests that space HZE particles with LET > 100 keV/μm could more effectively activate retrotransposons through demethylation in the F0 generation of space-exposed seeds (**Figure 3B**).

### 3.4 Genetic characteristics of Ty3-retrotransposons activated by spaceflight in offspring

Through the aforementioned research, it was found that spaceflight induced changes in the methylation levels of Ty3-retrotransposons in the rice genome. However, only when rice seeds were exposed to space HZE particles with LET > 100 keV/μm were retrotransposons more effectively activated through demethylation. To analyze the genetic variation patterns in progeny of Ty3-retrotransposons activated after spaceflight, the rice seeds of DN423 and DN416, from the same batch of flights as Nipponbare, were planted in conventional fields upon returning to the ground. In the F0 generation, rice plants that exhibited significant phenotypic changes after space radiation were identified and subsequently named SA3-7, SA6-2, and SC6-6. In order to ensure the simultaneous analysis of successive generations of bio-materials, 15 plants of SA3-7, SA6-2, and SC6-6 and corresponding ground control plants of the F2-F4 generations were planted in a phytotron at the same time. The F3-F5 generations of space mutagenic plants were simultaneously obtained and applied in this study (details in Materials and Methods).

The leaves of SA3-7, SA6-2, and SC6-6 and corresponding ground control were randomly selected per generation (n=3) and subjected to high-coverage (40×) whole-genome resequencing. The TIPs sites within the genomes of different samples were extracted using ITEIS (detailed in Materials and Methods). In this study, compared to their corresponding controls, the polymorphic changes in the insertions of the Ty3-retrotransposon families in the genomes of three individual plants from each mutant line were analyzed in the F3-F5 generations. Subsequently, the presence of these transposon insertion sites was examined in each individual of the F3 to F5 generations to determine whether these TIPs sites were stably inherited.

**Figure 4:**
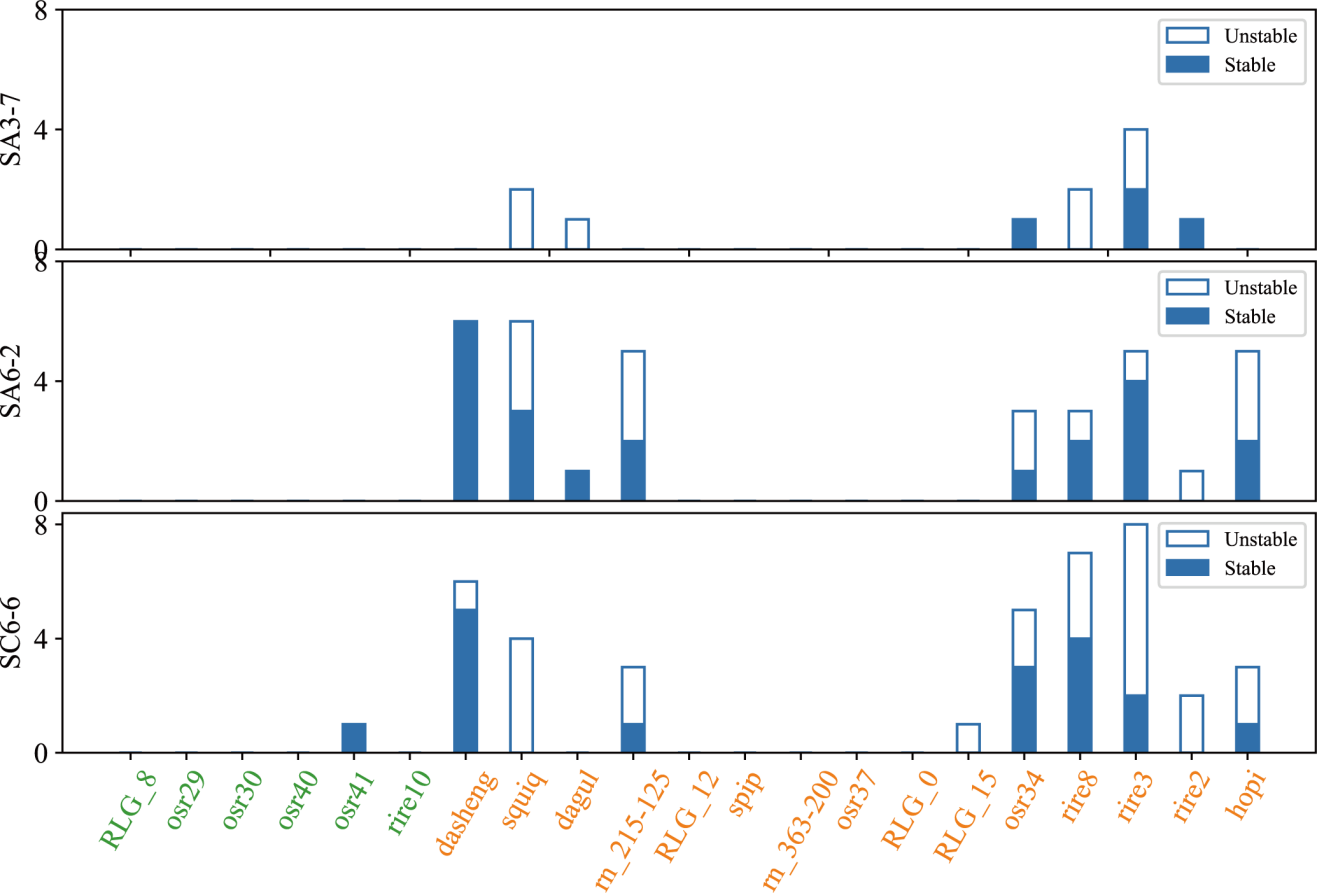
The number of stable and unstable inherited insertion sites in SA3-7, SA6-2, and SC6-6. The picture showed that the number of stable and unstable inherited insertion sites across F3 to F5 generations in SA3-7, SA6-2, and SC6-6. The x-axis represented different TE families, and the y-axis represented the space mutagenic lines SA3-7, SA6-2, and SC6-6. The blue bars indicated the number of stable inherited TE insertion sites, while the blank spaces indicated the number of unstable inherited TE insertion sites.

The results indicated that the TIPs of Ty3-retrotransposon induced after spaceflight mostly belonged to the young retrotransposons family, which included rire3, rire8, dasheng, osr34, hopi, squiq, rn_215-125, rire2, dagul and RLG_15. Only one ancient family element (osr41) was transposition in SC6-6 (**Figure 4**). The number of amplified Ty3-retrotransposons ranged from 1 to 8 in SA3-7, SA6-2, and SC6-6, and the insert sites of osr34 and rire2 in SA3-7, dasheng and dagul in SA6-2, and osr41 in SC6-6 were stably inherited between generations. Other Ty3-retrotransposons, such as rire3 and hopi, had some insertion sites in the F3-F5 generations of SA6-2, SA3-7, and SC6-6 that were not stably inherited.

**Figure 5:**
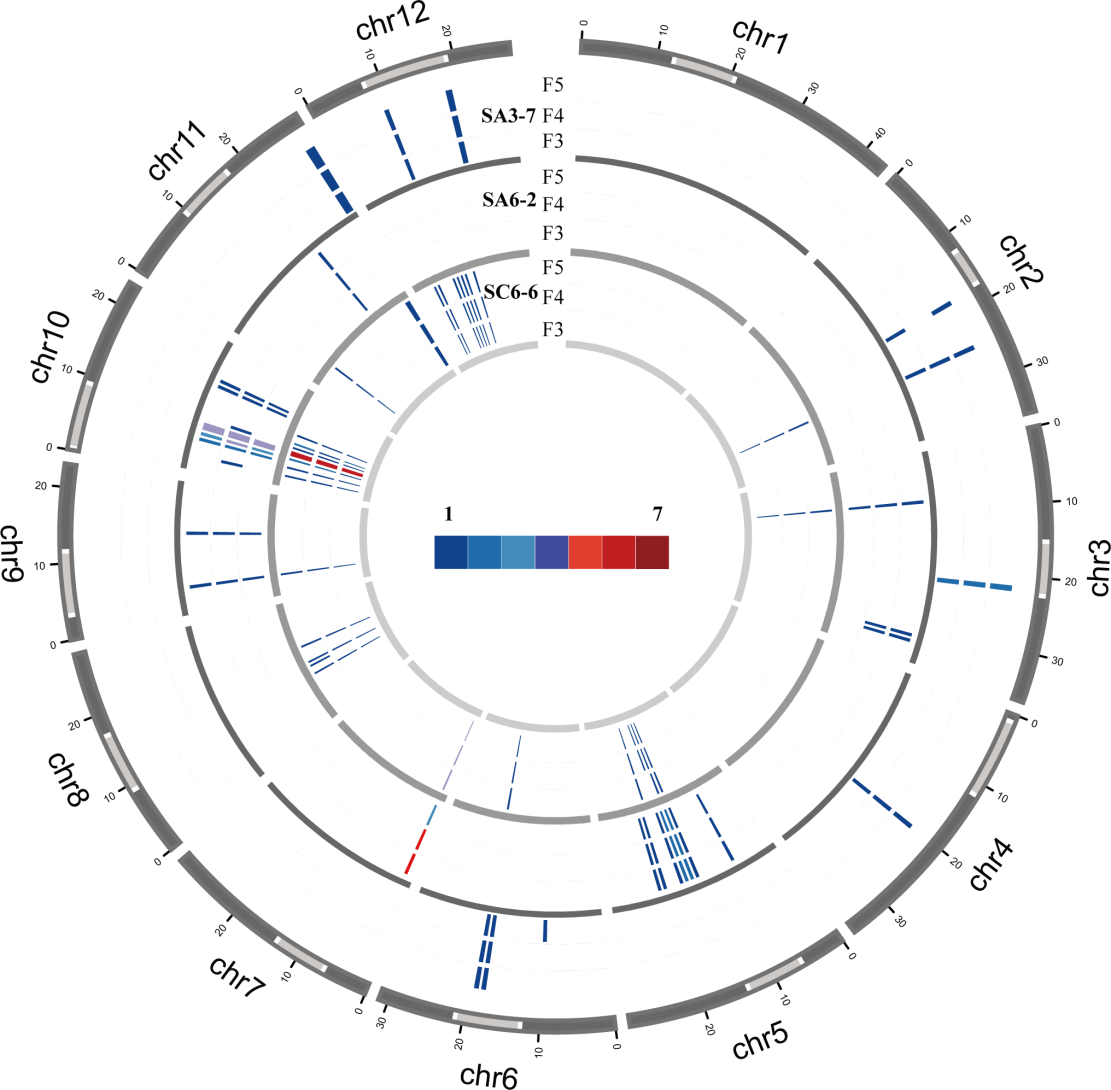
The number of TE insertion sites per bin (1,000,000 bp) in SA3-7, SA6-2, and SC6-6. The color gradient, ranging from blue to red, represented the quantity of TE insertions in each bin. The dark gray areas represented euchromatic regions on each chromosome, while the light gray areas represented heterochromatic regions.

This study further analyzed the distribution of TIPs sites on the chromosomes. In SA3-7, the insertion sites were distributed mainly on chromosomes 2, 3, 4, 6, 11 and 12. among them, 54.5% of the insertion sites were located in the heterochromatin region. In SA6-2, the insertions occurred mainly on chromosomes 3, 5, 7, 9, 10 and 11. Specifically, the insertions on chromosomes 5, 9, and 10 were detected in both the euchromatic and heterochromatic regions, while the remaining insertion sites were exclusively located in the euchromatic regions. Based on the results of Cheng et al., this study calculated the heterochromatin and euchromatin regions for each chromosome in the rice genome, as detailed in **Supplementary Table S5** (Cheng et al., 2001). By comparing the insertion sites of retrotransposons with different chromatin regions—euchromatin and heterochromatin—in the rice genome, this study found that in SA6-2, the insertion sites were preferentially inserted into the euchromatin region (62.9%). In SC6-6, the insertion sites were detected on all chromosomes except for chromosomes 1 and 4. Among them, 52.5% of the insertion sites preferred to insert into heterochromatin regions (**Figure 5**; **Supplementary Table S6**).

**Figure 6:**
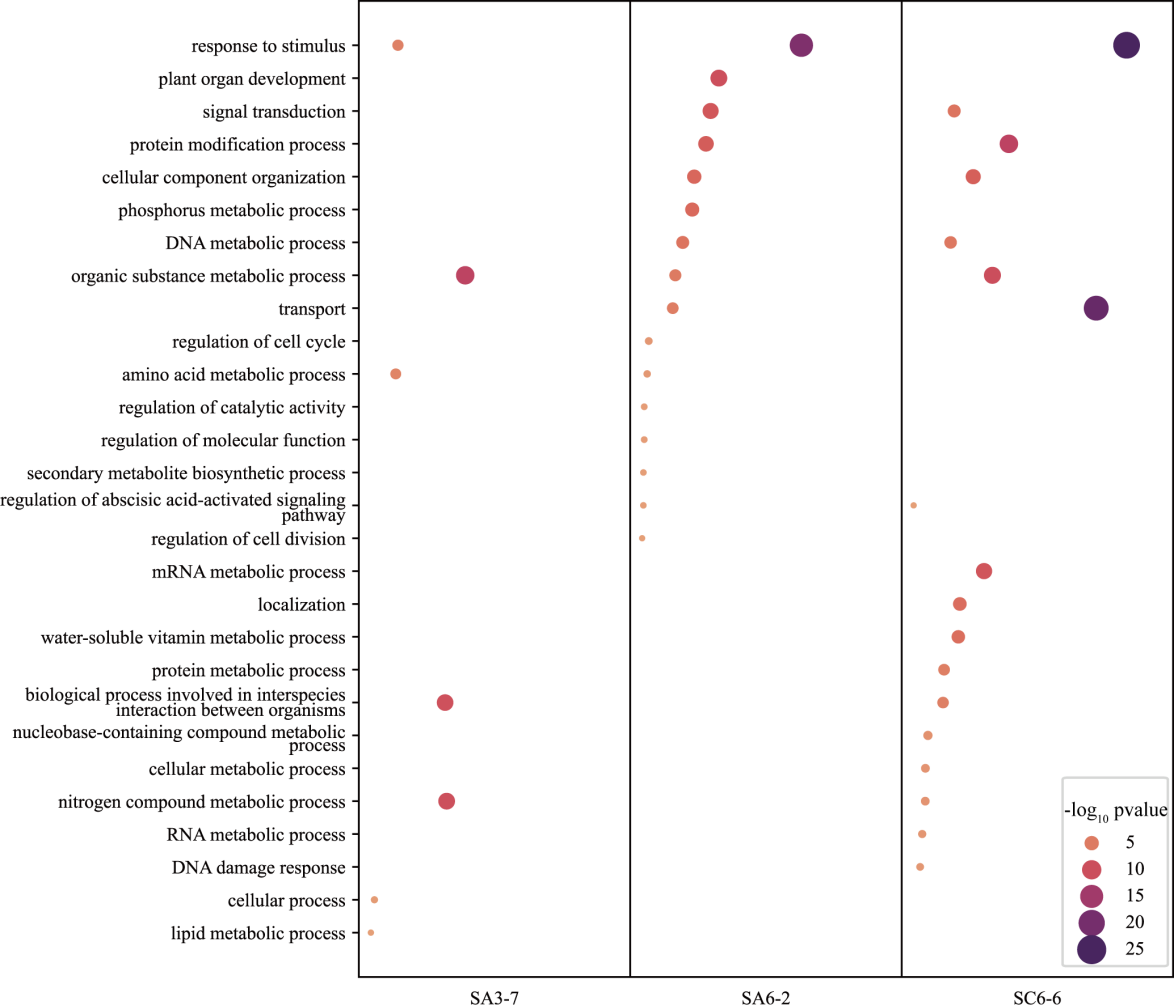
the GO enrichment analysis of genes proximal Ty3-retrotransposon insertion sites in space mutagenic lines SA3-7, SA6-2, and SC6-6.

In order to analyze the functional preference of Ty3-retrotransposon insertions, the genes of upstream and downstream in the insertion site were extracted and GO functional enrichment analysis was performed. Interestingly, The Ty3-retrotransposons always preferred to insert near genes related to the stress response and plant development (**Figure 6**). Those GO terms such as “response to stimulus”, “plant organ development” and “regulation of abscisic acid activation signaling pathways” were significantly enriched.

## 4 Discussion

Transposons played a significant role in angiosperm evolution and diversity and the content of transposons in plant genomes is closely related to genome size (Oliver and Greene, 2009; Oliver et al., 2011; Oliver and Greene, 2012; Keith et al., 2013). During stressful conditions, transposons may be activated and participate in genome remodeling and ultimately refining the host reaction to particular environmental stimuli (Slotkin and Martienssen, 2007; Hollister and Gaut, 2009; Feng et al., 2010; Zemach et al., 2010; Negi et al., 2016; Özgen et al., 2019). According to Oliver’s TE-Thrust hypothesis, the rapid expansion of transposons played a significant role in species evolution (Oliver and Greene, 2009; Oliver et al., 2011; Oliver and Greene, 2012; Keith et al., 2013). Space environment is a complex abiotic stress setting characterized by microgravity and strong radiation. The space radiation environment can induce genetic mutations in organisms within near-Earth orbit spacecraft (Yu et al., 2007; Long et al., 2009; Ou et al., 2009; Yatagai et al., 2011; Shi et al., 2014, 2017; Li et al., 2020; Jin et al., 2022). For example, it can alter cytosine methylation patterns and activate transposons (Ou et al., 2009; Xu et al., 2018). As humanity explores the space environment, understanding the mutagenesis patterns of plant genomes under space radiation and their hereditary characteristics in subsequent generations is of great significance.

### 4.1 Ty3-retrotransposons as powerful contributors to rice evolution

Transposons were a significant component of angiosperms, and the periodic burst amplification of retrotransposons within angiosperm genomes was closely related to their genome size (Daron et al., 2014; Sigman and Slotkin, 2016). In this study, the 1667 Ty3-retrotransposons of Nipponbare were divided into 176 transposon families, and those transposon families predominantly originated after 3.58 MYA. The TE families RLG_8, rire10, osr41, osr40, osr30, osr29 burst followed by gradual silencing around 2.6-0.78 MYA. Following 0.78 MYA, other transposons, including RLG_12, RLG_15, rire8, RLG_0, rire3, hopi, osr34, dagul, spip and osr37, underwent burst of transposition around 0.78-0 MYA and the number of transpositions was several times greater than that of the ancient transposons. The ancient transposons exhibit a higher methylation level than young transposons (**Figure 1C**), implying that they may have been effectively suppressed through strong methylation, making reactivation challenging. In addition, highly methylated Ty3-retrotransposon may be more likely to be removed from the host genome through recombination events (Choi and Purugganan, 2017).

### 4.2 Space HZE particles activated retrotransposons by demethylation

A wealth of studies has confirmed that plant seeds or seedlings exposed to spaceflight can induce changes in genome-wide DNA methylation levels, which play a role in regulating transposon activity. For example, Ou et al. found that after spaceflight, the DNA methylation levels of the transposable elements mPing, Tos17, Osr2, Osr23, Osr36, and Osr42 in the CG and CNG sequences of the rice genome were altered. Additionally, the DNA methylation patterns and expression states of these transposable elements were inherited by the offspring (Ou et al., 2009). Long et al. found that spaceflight induced the transposition activation of MITEs and LTR transposable elements in the rice genome by regulating cytosine methylation within these elements (Long et al., 2009). Shi et al. analyzed rice seeds exposed to spaceflight and low-dose heavy ion radiation and found that both spaceflight and low-dose heavy ion radiation induced significant changes in the epigenome of the rice genome. Moreover, in the CNG sequence context, CNG sequences were more prone to DNA methylation changes compared to CG sequences (Shi et al., 2014). And some studies discovered thousands of specifically methylated cytosines in the genome of Arabidopsis grown on the ISS, with significant organ-specific differences in methylation patterns (Xu et al., 2018; Zhou et al., 2019). In summary, these studies indicate that after spaceflight, the DNA methylation levels in the genomes of space-exposed plants undergo significant changes, and these DNA methylation changes play a role in regulating transposon activity within the genome. Therefore, a systematic analysis of the activity changes of complete LTR-RTs in the rice genome after spaceflight from a whole-genome perspective is of great significance for understanding the impact of space high-energy heavy ions on rice genome evolution after spaceflight. In addition, The burst expansions of retrotransposon was a powerful drivers to angiosperm evolution (Keith et al., 2013). Therefore, exploring the relationship between space HZE particles and retrotransposon activity was of great significance.

This study found that the rice seeds after being hit by space HZE particles with different LET, both ancient and young Ty3-retrotransposons showed changes in methylation for the F0 generation of space-exposed seeds compared to their controls. There was a dynamic shift from hyper-methylation to demethylation, from the tillering stage to the heading stage, especially in young transposons, when hit by HZE particles with LET ≥ 100 keV/μm. These demethylation changes induced changes in transcription of retrotransposon, and the number of demethylation and increased transcription retrotransposons of dagul, spip, osr37, RLG_0, RLG_15, osr34, rire8, rire3, rire2, and hopi were significantly higher when rice seeds were hit by space HZE particles with LET > 100 keV/μm. Among them, the number of transposons with increased transcription levels in rire3, rire2, and hopi ranged from 24 to 43. This suggests that space HZE particles with LET > 100 keV/μm could more effectively activate retrotransposons from families such as dagul, spip, osr37, RLG_0, RLG_15, osr34, rire8, rire3, rire2, and hopi in the F0 generation of space-exposed seeds through demethylation.

### 4.3 Genetic characteristics of Ty3-retrotransposons activated after spaceflight

Transposons were a significant component of angiosperm genomes, and their abundance was closely correlated with the genome size of angiosperms (Negi et al., 2016). Oliver et al. found that the amplification and evolution of transposons were closely linked to the evolution and diversity of angiosperms. They proposed that TE-Thrust promoted genomic plasticity, providing a more comprehensive and satisfactory explanation for Darwin’s ‘abominable mystery’ (Keith et al., 2013).

In order to investigate the stability of the insertion sites of activated Ty3-retrotransposons after spaceflight in subsequent generations, in this study, the TIPs in the F3 to F5 generations of the SA3-7, SA6-2, and SC6-6 mutant lines were analyzed. Younger TE families, including rire3, rire8, dasheng, osr34, hopi, squiq, rn_215-125, rire2, dagul and RLG_15, exhibited transposon insertions in subsequent generations. However, the ancient transposon families exhibited virtually no transposon insertions. The result indicated that although ancient transposons can also undergo demethylation and transcription, they did not transpose in subsequent generations. Additionally, the study found that rire8, squiq, and dagul in SA3-7; rire2 in SA6-2; and rire2 and RLG_15 in SC6-6 were not stably inherited from the F3 to F5 generations. It indicated that these transposon families still exhibit polymorphic changes in subsequent generations. For example, the transposon insertions of osr34 and rire2 in SA3-7, dasheng and dagul in SA6-2, and osr41 in SC6-6 were stably inherited between the F3 and F5 generations. Other families with transposon insertions, such as rire3 and hopi had some insertion sites in the F3-F5 generations of different mutant lines that were not stably inherited.

The transposon insertion sites in the genomes of SA3-7, SA6-2 and SC6-6 revealed that more than half of the insertions occurred in heterochromatin regions. RNA retrotransposons were primarily located in centromeric regions (Lippman et al., 2004; Dawe and Henikoff, 2006; Bennetzen et al., 2012; Tsukahara et al., 2012; Bennetzen and Wang, 2014; Sigman and Slotkin, 2016). The insertion of RNA retrotransposons could lead to chromosomal instability and potentially cause non-disjunction or mis-segregation of chromosomes, resulting in abnormal cell division (Burrack and Berman, 2012). In addition, the transposon families rire3, rire8, and osr34 in the SA3-7, SA6-2, and SC6-6 mutants inserted into euchromatin regions after activation (detailed in **Supplementary Table S6**). The analysis of genes upstream and downstream of the insertion sites in functional gene regions revealed that Ty3-retrotransposons consistently preferred to insert into genes related to stress response and plant development (**Figure 6**). Those GO terms such as “response to stimulus”, “plant organ development” and “regulation of abscisic acid activation signaling pathways” were significantly enriched.

### 4.4 Space HZE particles might have been a trigger for the burst expansion of angiosperms

In the space radiation environment, ionizing radiation from various sources, such as heavy ions, protons, and electrons, can affect biological organisms. Based on their sources, radiation is classified into three types: Galactic Cosmic Rays (GCR), Solar Particle Events (SPE), and Trapped Belt Radiation. Due to the protection of the Earth by a nearly spherical geomagnetic field, terrestrial organisms are shielded from the effects of space radiation. The Earth’s magnetic field consists of a dipole magnetic field and a non-dipole magnetic field, with a magnetic field strength ranging from 25 to 65 µT. The dipole magnetic field is characterized by magnetic field lines that extend from the north pole to the south pole, creating an approximate dipole configuration. It accounts for approximately 80-90% of the total magnetic field (Finlay et al., 2010). Since the magnetosphere is located thousands of kilometers from the Earth, the Earth’s magnetic field (GMF) can influence the spatial radiation. It is precisely due to the presence of the Earth’s magnetic field that most charged particle flows in space are deflected, thereby protecting the Earth’s biosphere from the effects of space radiation such as solar wind (a stream of high-energy charged particles emitted by the Sun) (Occhipinti et al., 2014; Tarduno et al., 2015).

Geomagnetic field (GMF) reversal occurred approximately every few million years in the evolutionary history of the Earth (Leonardo et al., 2016; Kulakov et al., 2019; Griffing et al., 2022; Pan and Li, 2023). The intensity of the dipole field decreased to 10% of the original strength during GMF reversal (Pan and Li, 2023). Occhipinti et al. reported that the periods of normal polarity transitions of GMF overlapped with the diversion of most often familial angiosperm lineage (Occhipinti et al., 2014). This suggested that the widespread proliferation of angiosperms might have been associated with geomagnetic reversals. Aside from the above mentioned studies, hicxey et al. reported that new forms of animals and plants first appeared in the Arctic and subsequently migrated to temperate climes (Hickey et al., 1983). And owing to the shelter provided by water, a gradually slower speciation evolutionary rate would be expected moving from land to shallow water to deep water (Jablonski et al., 1983; Buzas and Culver, 1984). This suggested that species diversification was more prevalent in areas of the Earth’s surface that were relatively exposed to cosmic radiation and indicated a possible connection between geomagnetic reversals and the burst expansion of angiosperms.

In this study, we found that the burst expansions of ancient and young Ty3-retrotransposons in rice occurred after 2.6 and 0.78 MYA, respectively. These timings corresponded precisely to the Gauss–Matuyama and Brunhes–Matuyama GMF reversals, which occurred approximately 2.6 and 0.78 MYA. Moreover, this study found space HZE particles with LET > 100 keV/μm could more effectively activate retrotransposons through demethylation in young retrotransposons in the F0 generation of space-exposed seeds. And the retrotransposons showing polymorphic insertions predominantly belonged to the younger Ty3-retrotransposons in the F3 to F5 generations of SA3-7, SA6-2, and SC6-6. During GMF reversals, Earth’s organisms encounter not only decreased GMF strength and increased exposure to ultraviolet radiation but also experience significantly reduced shielding against space HZE particles. Consequently, space HZE particles may have activated Ty3-retrotransposons within the genome of angiosperm, potentially influencing their evolution during GMF reversals.

## 5 Conclusion

This study found that the Ty3-retrotransposon families experienced two explosive burst expansions at 2.6 - 0.78 MYA (ancient) and 0.78 - 0 MYA (young). When rice seeds were exposed to space HZE particles with LET > 100 keV/μm, the ancient transposon families (osr30, osr40, and rire10) and young transposon families (dagul, rn215-125, osr37, RLG_15, osr34, rire8, rire3, rire2, and hopi) were activated in the F0 generation of space-exposed seeds. Additionally, these activated transposons activated after spaceflight also showed insertion polymorphisms in the genome of F3-F5 successive generations in DN416 and DN423 mutants induced by spaceflight, and most of these insertions tended to be stably inherited.

## Data Availability

The datasets presented in this study can be found in online repositories. The names of the repository/repositories and accession number(s) can be found below: https://ngdc.cncb.ac.cn/gsa, CRA013907, CRA013933, CRA013870, and CRA013651.

## Supplementary material

Supplementary Figure 1 Sampling diagram of space mutagenetic lines. Space mutagenic plants were identified in individuals of F0 generation. Plants mutated by space radiation, which exhibited changes in plant height and tillering number, were selected and named SA3-7, SA6-2 and SC6-6 respectively. The offspring were tracked and planted using pedigree method. Three successive generations of material were simultaneously planted in the phytotron. F3-F5 generation of space mutagenic plants were utilized in this research.

Supplementary Figure 2 Methods of identifying transposable elements (TEs) insertion sites. the reads from resequencing were aligned to the reference genome, and the result can appear in two forms: **(A)** which indicated the presence of a new insertion site within the target site duplication (TSD) domain, and **(B)** which represented the normal alignment with no sequence insertion. the sequences corresponding to the positive or negative strands of the soft-clip sequences from A were extracted. Subsequently, these sequences were aligned to TEs in **(C)**. The transposon might have inserted into a new site if the sequences aligned to both ends of the TEs.

Supplementary Figure 3 The number of Ty3-retrotransposons with increased transcription across different groups and transposon families during the tillering and heading stages.
