# Supplementary figures and images for: Activation characteristics of Ty3-retrotransposons after spaceflight and genetic stability of insertion sites in rice progeny
Source: Front Plant Sci. 2024 Dec 2;15:1452592. doi: 10.3389/fpls.2024.1452592 (PMC11646775; doi:10.3389/fpls.2024.1452592)

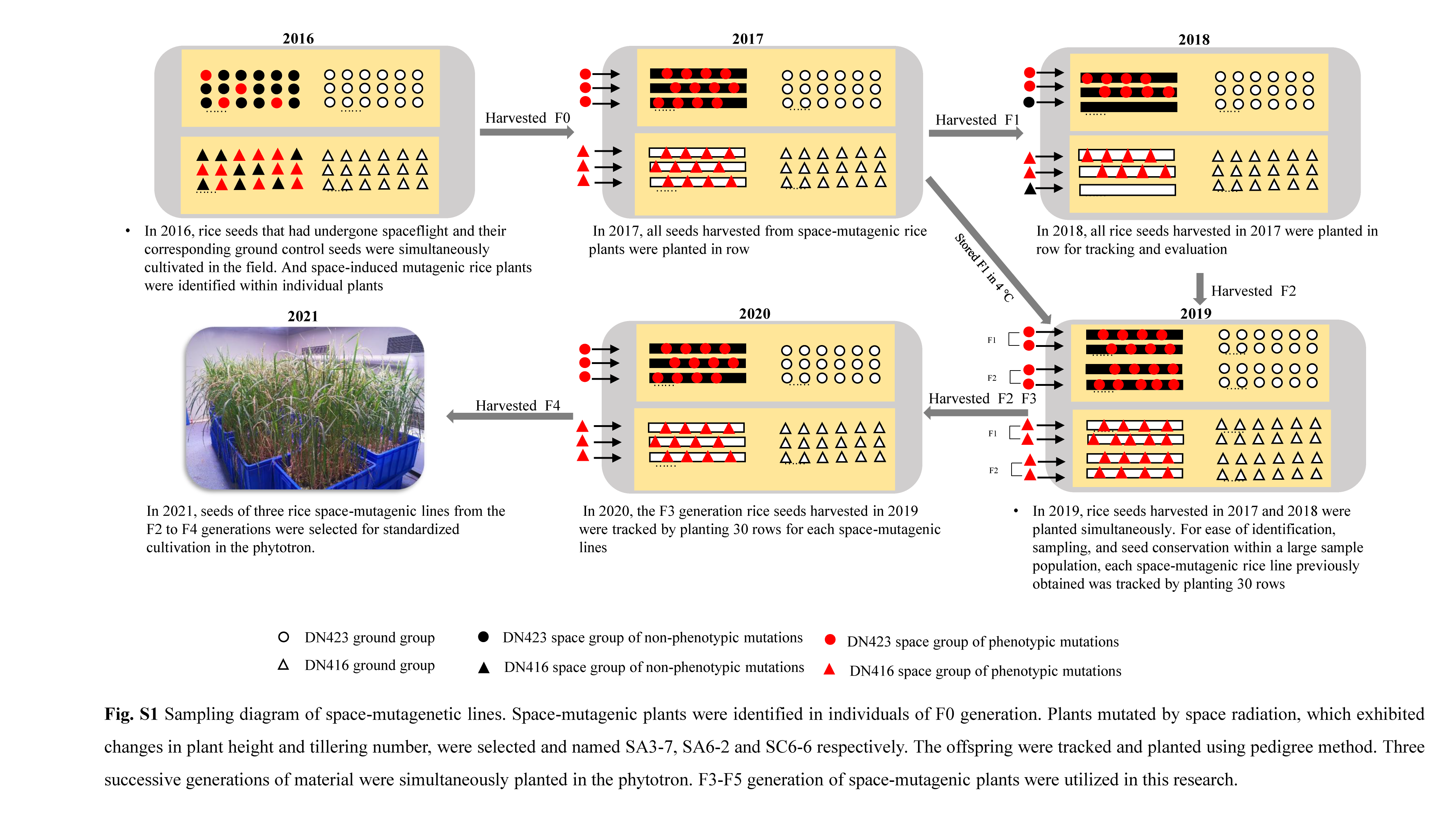

Supplement: Supplementary Figure 1 — Sampling diagram of space mutagenetic lines. Space mutagenic plants were identified in individuals of F0 generation. Plants mutated by space radiation, which exhibited changes in plant height and tillering number, were selected and named SA3-7, SA6-2 and SC6-6 respectively. The offspring were tracked and planted using pedigree method. Three successive generations of material were simultaneously planted in the phytotron. F3-F5 generation of space mutagenic plants were utilized in this research. [file Image1.tif]

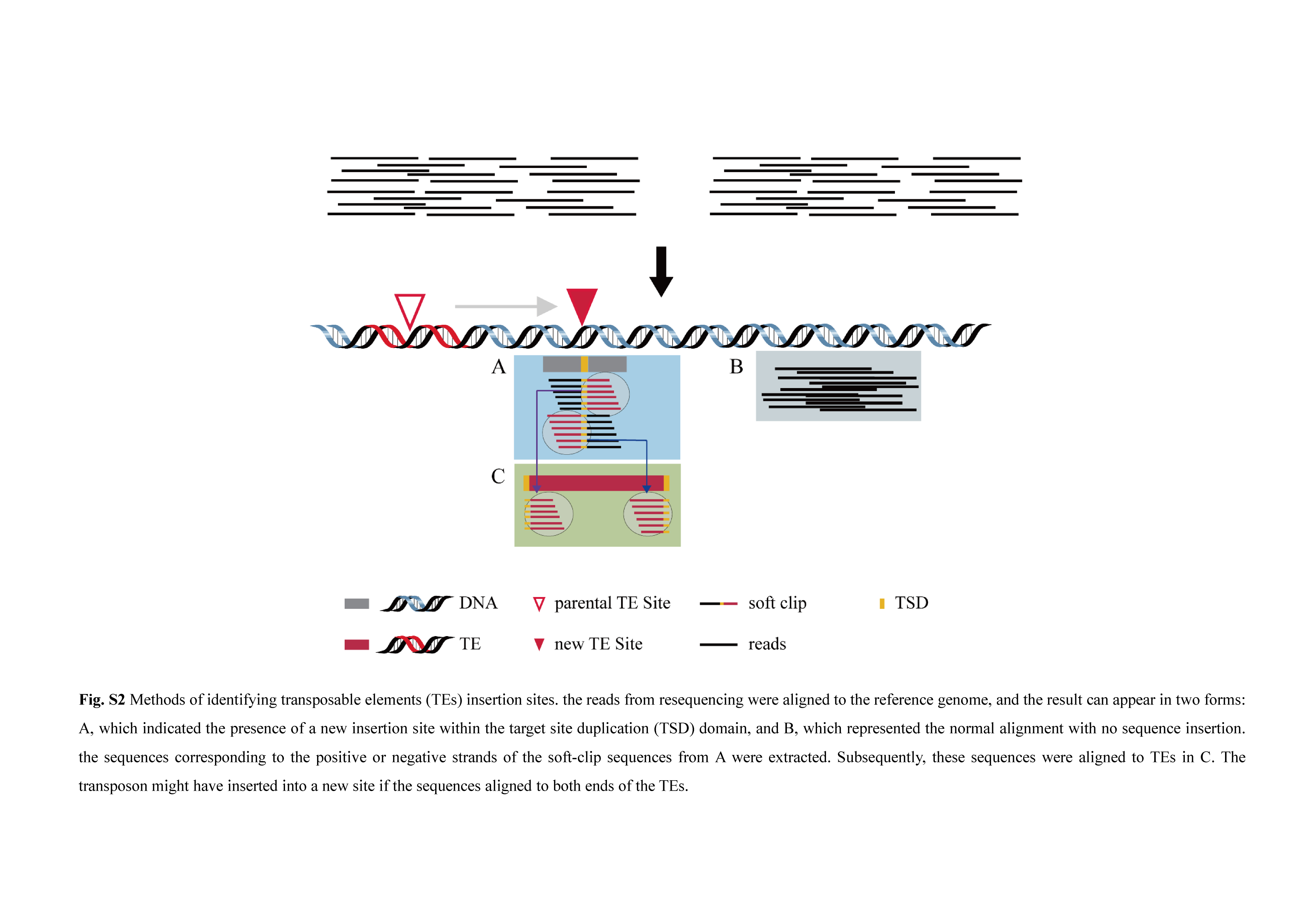

Supplement: Supplementary Figure 2 — Methods of identifying transposable elements (TEs) insertion sites. the reads from resequencing were aligned to the reference genome, and the result can appear in two forms: (A) which indicated the presence of a new insertion site within the target site duplication (TSD) domain, and (B) which represented the normal alignment with no sequence insertion. the sequences corresponding to the positive or negative strands of the soft-clip sequences from A were extracted. Subsequently, these sequences were aligned to TEs in (C). The transposon might have inserted into a new site if the sequences aligned to both ends of the TEs. [file Image2.tif]

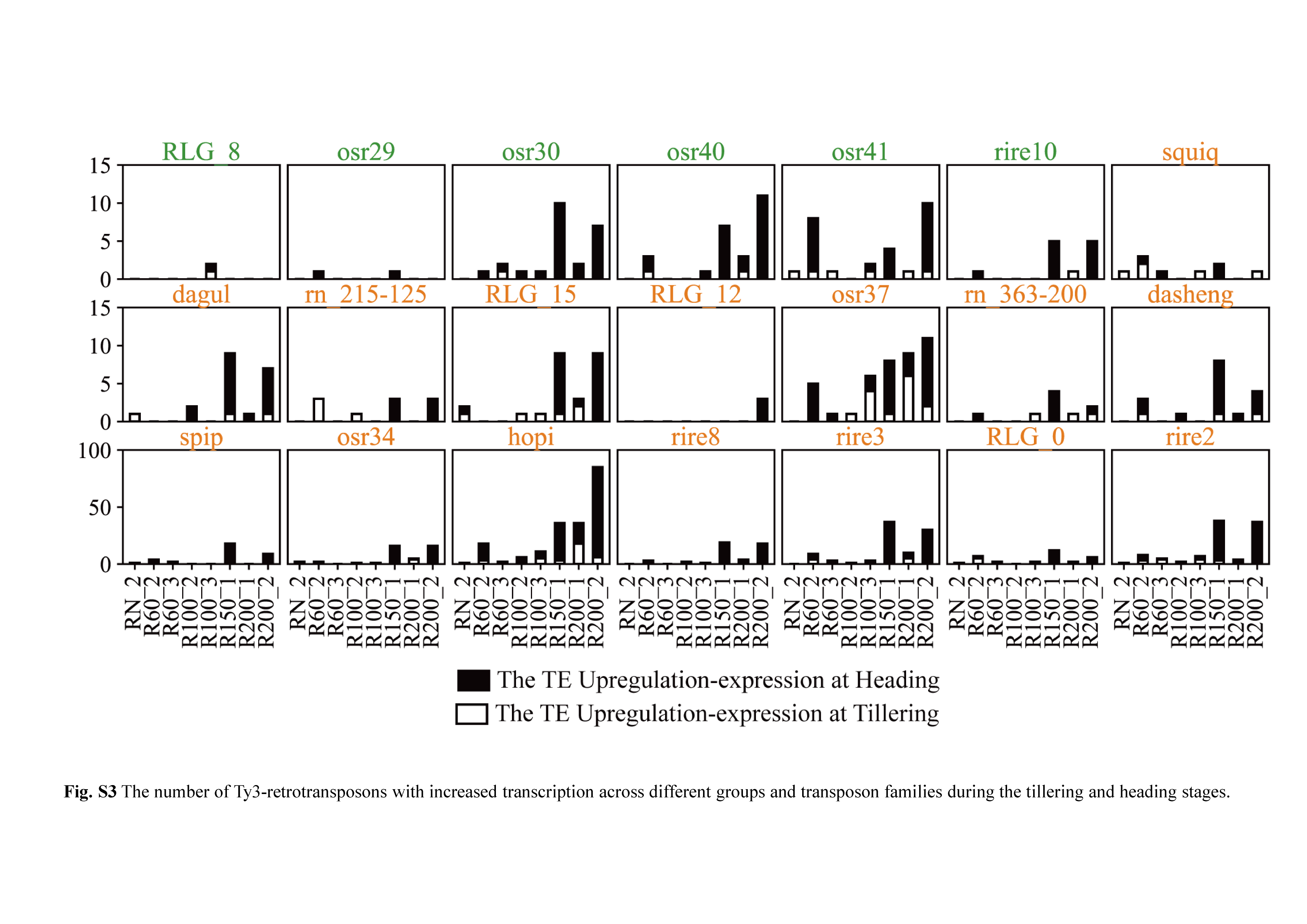

Supplement: Supplementary Figure 3 — The number of Ty3-retrotransposons with increased transcription across different groups and transposon families during the tillering and heading stages. [file Image3.tif]
